# Rigorous antibiotic stewardship in the hospitalized elderly population: saving lives and decreasing cost of inpatient care

**DOI:** 10.1093/jacamr/dlab118

**Published:** 2021-08-12

**Authors:** James Mauro, Saman Kannangara, Joanne Peterson, David Livert, Roman A Tuma

**Affiliations:** 1 Department of Pharmacy, Easton Hospital, Easton, PA, USA; 2 Department of Internal Medicine, Division of Infectious Diseases, Saint Francis Memorial Hospital, San Francisco, CA, USA; 3 Department of Infection Control, Hackensack Meridian, Bayshore Medical Center, Holmdel, NJ, USA; 4 Department of Medicine, Easton Hospital, Easton, PA, USA; 5 Penn State University, Center Valley, PA, USA; 6 Department of Internal Medicine, Hackensack Meridian, Bayshore Medical Center, Holmdel, NJ, USA; 7 Department of Medicine, Hackensack Meridian School of Medicine, Nutley, NJ, USA

## Abstract

**Background:**

There is limited literature evaluating the effect of antibiotic stewardship programmes (ASPs) in hospitalized geriatric patients, who are at higher risk for readmissions, developing *Clostridioides difficile* infection (CDI) or other adverse outcomes secondary to antibiotic treatments.

**Methods:**

In this cohort study we compare the rates of 30 day hospital readmissions because of reinfection or development of CDI in patients 65 years and older who received ASP interventions between January and June 2017. We also assessed their mortality rates and length of stay. Patients were included if they received antibiotics for pneumonia, urinary tract infection, acute bacterial skin and skin structure infection or complicated intra-abdominal infection. The ASP team reviewed patients on antibiotics daily. ASP interventions included de-escalation of empirical or definitive therapy, change in duration of therapy or discontinuation of therapy. Treatment failure was defined as readmission because of reinfection or a new infection. A control group of patients 65 years and older who received antibiotics between January and June 2015 (pre-ASP) was analysed for comparison.

**Results:**

We demonstrated that the 30 day hospital readmission rate for all infection types decreased during the ASP intervention period from 24.9% to 9.3%, *P *<* *0.001. The rate of 30 day readmissions because of CDI decreased during the intervention period from 2.4% to 0.30%, *P *=* *0.02. Mortality in the cohort that underwent ASP interventions decreased from 9.6% to 5.4%, *P *=* *0.03. Lastly, antibiotic expenditure decreased after implementation of the ASP from $23.3 to $4.3 per adjusted patient day, in just 6 months.

**Conclusions:**

Rigorous de-escalation and curtailing of antibiotic therapies were beneficial and without risk for the hospitalized patients 65 years and over.

## Introduction

More than 50% of patients in the USA receive one or more antibiotic treatments during their hospital stay, and MDR bacteria are ever increasing.^1^ The 2019 Antibiotic Threats Report by the CDC stated that more than 2.8 million antibiotic resistant infections occur in the USA each year, and more than 35 000 people died as a result. In 2017, nearly 223 900 people required hospitalization in the USA because of *Clostridioides difficile* infections (CDIs), and at least 12 800 people died thereof. The CDC has categorized CDIs as an urgent threat.^2^ Most of the CDIs occur in the elderly population and cost the USA an estimated $8 billion annually.^3^ The elderly are the most vulnerable patient population because of immune system senescence and multiple comorbidities as well as polypharmacy, leading to multiple drug–drug interactions, and increasing the risk of MDR infections.^4^^,^^5^ In fact, by 2050, approximately 20% of the US population will be 65 years and older, and over the first 10 years of this decade antibiotic usage increased by 30% in the elderly.^6^ Therefore, geriatric patients should benefit the most from stringent antibiotic usage oversight.^7–9^ This is supported by the overall contribution of antibiotic stewardship programmes (ASPs) in decreasing hospital infections and MDR colonization, as well reduction of CDIs.^10^

The impact of ASP efforts on quality metrics remains a focus of investigations. Recent studies suggested that ASPs benefitted mortality reductions, in-hospital length of stay (LOS) reductions and a decrease in cost of care as accounted for by cost of antibiotic consumption.^11–13^ The effect of ASPs on readmission rates has not been frequently reported and was variable, ranging from favourable, to having no impact, to correlating with an increase in readmission rates.^11–14^

In this study, we investigated the impact of a rigorous ASP on quality metrics in a hospitalized, elderly population. The primary objective was to determine if our ASP decreased 30 day hospital readmissions secondary to reinfection, including readmissions because of CDIs. The secondary objective was to determine if the patient-specific infection diagnosis of pneumonia (PNA), urinary tract infection (UTI), acute bacterial skin and skin structure infection (ABSSSI) or complicated intra-abdominal infection (cIAI) influenced the effectiveness of the ASP interventions in preventing hospital readmissions. Moreover, would these efforts decrease LOS and in-hospital mortality, as well as the cost of hospitalization because of a reduction in antibiotic expenditure?

## Methods

This cohort study represents a single centre, retrospective chart review in a 256 bed teaching hospital in the USA. The ASP intervention group consisted of adult patients ≥65 years old, who received ASP interventions between January and June 2017. The control group consisted of adult patients ≥65 years old, who received antibiotic therapies between January and June 2015 (pre-ASP era).

Inclusion criteria included adult patients ≥65 years old, who received antibiotics during inpatient hospital stay and carried a diagnosis of PNA, UTI, ABSSSI or cIAI. Exclusion criteria included patients who died or transitioned to hospice care as well as non-acceptance of ASP recommendations in the intervention group. Statistical analyses employed *t*-test and χ^2^ statistics as well as Mann–Whitney *U* test where appropriate. Two-tailed *P* values less than 0.05 were considered statistically significant. We applied the Statistical Package R Core Team, IBM SPSS Version 27.0.1.0 (2020).^15^

All antibiotic therapy recommendations were based on clinical practice guidelines of IDSA, the American Thoracic Society and the Surgical Infection Society, and were implemented during the first 24–48 h of the initiation of antibiotic regimens. The following ASP interventions were conducted: (i) de-escalation or escalation of empirical or definitive therapy; (ii) change in duration of therapy; and (iii) discontinuation of therapy. Infectious diseases diagnoses were reviewed and appropriateness of antibiotics as well as dose adjustments were addressed within categories (i)–(iii). The ASP team consisted of a clinical pharmacist, an infectious diseases physician and a clinical nurse. Specifically, on a daily basis the clinical pharmacist, after consulting with the ASP infectious disease specialist, called the respective attending physicians and conveyed antibiotic treatment recommendations. The following day, the patient’s medical records were reviewed to assess adherence to recommendations. If recommendations were not followed, the ASP conducted a re-review, and the infectious disease specialist personally contacted the antibiotic prescriber attending for discussion and follow through. Daily review of preliminary and final microbiological culture results was part of the decision-making but pending results did not preclude intervention recommendations. Education of the medical staff was an integral part of the ‘buy-in’ and was conducted by one-on-one phone conversations between the clinical pharmacist or infectious disease specialist and the antibiotic prescribing physician. Of note, our actual ASP was started in the last quarter of 2015. Otherwise, we did not identify any changes in the clinical decision-making systems supporting antibiotic prescribing, such as our electronic healthcare record, nor were approval processes for prescribing antibiotics changed for the control and intervention group, beyond the ASP recommendations. General medical management principles, including infectious diseases consulting practices and readmission initiatives, as well as the overall demographics of the admitted elderly patients did not undergo any apparent changes and were comparable during the control and intervention periods of this study. Our readmission rates reflected returns to the hospital within 30 days after initial discharge because of reinfection or new infection. Readmissions secondary to other comorbidities, accounting for the overall readmission rate, were not included. The mortality rates represented in-hospital mortalities.

### Ethics

This study’s protocol was approved by the Institutional Review Board (IRB) of Easton Hospital, Easton, PA, USA. All personal information of patients was maintained confidentially. Because we used retrospective data retrieved from medical records based on IRB permission, patients’ informed consent was not obtained.

## Results

Table 1 outlines the demographics and infection types as well as the overall utilization of antibiotics and their cost in the ASP-intervention group (pre- and post-review) and the historical control group. Antibiotics utilized in less than five patient regimens were grouped into the ‘other’ category. Overall adherence to ASP recommendations was >95%. Of note, the majority of ASP interventions were de-escalations (62%) and discontinuations (24%) (Table 2). These interventions resulted in a remarkable decrease of broad-spectrum antibiotic therapies as well as glycopeptide utilization (Table 1). Conversely, first-generation cephalosporin and penicillin as well as aminopenicillin antibiotics were not administered in the intervention group prior to ASP recommendation nor in the historical control group (Table 1). Antibiotic utilization by infection type is detailed in Table 3.

**Table 1.** Patient characteristics

Characteristic	Control (*n = *544)	ASP intervention (*n = *297)		*P* value
		pre-review	post-review	
Age, years, mean	79.5	80.2		0.26
Male gender, *n* (%)	239 (44.0)	138 (46.6)		0.48
Infection type, *n* (%)				
PNA	268 (49.4)	135 (45.5)		0.27
UTI	132 (24.4)	100 (33.7)		0.005
ABSSSI	83 (15.3)	35 (11.8)		0.026
cIAI	61 (11.3)	14 (4.7)		0.001
other^a^	13 (2.4)	13 (4.4)		0.111
Antimicrobial therapies, *n* (%)^b^				
carbapenem	80 (9.2)	12 (4.0)	1 (0.5)	—
penicillin (antipseudomonal)	161 (18.6)	72 (24.1)	8 (3.7)	—
cephalosporin (fourth generation)	91 (10.5)	35 (11.7)	8 (3.7)	—
fluoroquinolone	116 (13.4)	6 (2.0)	12 (5.6)	—
cephalosporin (third generation)	71 (8.2)	43 (14.4)	49 (22.9)	—
penicillin (aminopenicillin)	0 (0.0)	2 (0.7)	35 (16.4)	—
cephalosporin (fifth generation)	10 (1.2)	0 (0.0)	0 (0.0)	—
monobactam	28 (3.2)	4 (1.3)	0 (0.0)	—
cephalosporin (first generation)	0 (0.0)	0 (0.0)	52 (24.3)	—
miscellaneous^c^	27 (3.1)	13 (4.3)	22 (10.3)	—
glycopeptide	253 (29.2)	93 (31.1)	17 (7.9)	—
oxazolidinone	9 (1.0)	2 (0.7)	2 (0.9)	—
other^d^	20 (2.3)	6 (2.0)	8 (3.7)	—
Antimicrobial expenditure, $				
total	379 643	67 721		—
cost/APD	23.33	4.37		—

a Sepsis, fever (neutropenic, post-operative, unknown origin), osteomyelitis, prosthetic joint infection, leucocytosis.

b Antimicrobials used as monotherapy or in combination with other agents.

c Fosfomycin, metronidazole, nitrofurantoin, trimethoprim/sulfamethoxazole.

d Azithromycin, clindamycin, daptomycin, doxycycline, fluconazole, micafungin, tigecycline.

**Table 2.** Type of ASP intervention

Intervention type	*n* (%)
De-escalation	185 (62.3)
Discontinuation	71 (23.9)
Duration	30 (10.1)
Escalation	12 (4.0)

**Table 3.** Antimicrobial usage by infection type^a^

Infection type/Antimicrobial	Control	ASP intervention	
		pre-review	post-review
PNA, *n* (%)			
carbapenem	35 (8.1)	3 (2.4)	1 (1.1)
penicillin (antipseudomonal)	83 (19.3)	42 (33.6)	3 (3.4)
cephalosporin (fourth generation)	27 (6.3)	13 (10.4)	5 (5.6)
fluoroquinolone	53 (12.3)	2 (1.6)	5 (5.6)
cephalosporin (third generation)	53 (12.3)	4 (3.2)	32 (35.9)
penicillin (aminopenicillin)	3 (0.7)	0 (0.0)	15 (16.8)
cephalosporin (fifth generation)	1 (0.2)	0 (0.0)	0 (0.0)
monobactam	17 (4.0)	4 (3.2)	0 (0.0)
cephalosporin (first generation)	0 (0.0)	1 (0.8)	5 (5.6)
miscellaneous^b^	1 (0.2)	9 (7.2)	13 (14.6)
glycopeptide	146 (34.0)	46 (36.8)	5 (5.6)
oxazolidinone	2 (0.5)	0 (0.0)	0 (0.0)
other^c^	9 (2.1)	2 (1.6)	5 (5.6)
UTI, *n* (%)			
carbapenem	19 (10.5)	7 (9.2)	0 (0.0)
penicillin (antipseudomonal)	30 (16.6)	6 (7.9)	0 (0.0)
cephalosporin (fourth generation)	17 (9.4)	8 (10.5)	1 (1.6)
fluoroquinolone	29 (16.0)	3 (3.9)	4 (6.3)
cephalosporin (third generation)	35 (19.3)	39 (51.3)	3 (4.7)
penicillin (aminopenicillin)	0 (0.0)	1 (1.3)	10 (15.6)
cephalosporin (fifth generation)	0 (0.0)	0 (0.0)	0 (0.0)
monobactam	6 (3.3)	0 (0.0)	0 (0.0)
cephalosporin (first generation)	0 (0.0)	0 (0.0)	38 (59.4)
miscellaneous^b^	0 (0.0)	1 (1.3)	4 (6.3)
glycopeptide	41 (22.7)	8 (10.5)	2 (3.1)
oxazolidinone	4 (2.2)	2 (2.6)	1 (1.6)
other^c^	3 (1.7)	1 (1.3)	1 (1.6)
ABSSSI, *n* (%)			
carbapenem	12 (9.2)	1 (2.0)	0 (0.0)
penicillin (antipseudomonal)	26 (20.0)	11 (22.0)	2 (7.4)
cephalosporin (fourth generation)	6 (4.6)	7 (14.0)	1 (3.7)
fluoroquinolone	10 (7.7)	1 (2.0)	2 (7.4)
cephalosporin (third generation)	1 (0.8)	2 (4.0)	4 (14.8)
penicillin (aminopenicillin)	1 (0.8)	3 (6.0)	3 (11.1)
cephalosporin (fifth generation)	9 (6.9)	0 (0.0)	0 (0.0)
monobactam	2 (1.5)	0 (0.0)	0 (0.0)
cephalosporin (first generation)	0 (0.0)	0 (0.0)	7 (25.9)
miscellaneous^b^	0 (0.0)	2 (4.0)	1 (3.7)
glycopeptide	58 (44.6)	21 (42.0)	5 (18.5)
oxazolidinone	1 (0.8)	0 (0.0)	0 (0.0)
other^c^	4 (3.1)	2 (4.0)	2 (7.4)
cIAI, *n* (%)			
carbapenem	12 (11.5)	1 (11.1)	0 (0.0)
penicillin (antipseudomonal)	20 (19.2)	4 (44.4)	2 (22.2)
cephalosporin (fourth generation)	2 (1.9)	1 (11.1)	0 (0.0)
fluoroquinolone	22 (21.1)	0 (0.0)	0 (0.0)
cephalosporin (third generation)	1 (1.0)	0 (0.0)	3 (33.3)
penicillin (aminopenicillin)	0 (0.0)	0 (0.0)	1 (11.1)
cephalosporin (fifth generation)	0 (0.0)	0 (0.0)	0 (0.0)
monobactam	2 (1.9)	0 (0.0)	0 (0.0)
cephalosporin (first generation)	0 (0.0)	0 (0.0)	0 (0.0)
miscellaneous^b^	25 (24.0)	1 (11.1)	3 (33.3)
glycopeptide	14 (13.5)	2 (22.2)	0 (0.0)
oxazolidinone	2 (1.9)	0 (0.0)	0 (0.0)
other^c^	4 (3.8)	0 (0.0)	0 (0.0)
Other, *n* (%)^d^			
carbapenem	2 (10.0)	0 (0.0)	0 (0.0)
penicillin (antipseudomonal)	2 (10.0)	9 (23.1)	1 (4.0)
cephalosporin (fourth generation)	5 (25.0)	9 (23.1)	1 (4.0)
fluoroquinolone	2 (10.0)	1 (2.6)	1 (4.0)
cephalosporin (third generation)	1 (5.0)	1 (2.6)	7 (28.0)
penicillin (aminopenicillin)	0 (0.0)	1 (2.6)	6 (24.0)
cephalosporin (fifth generation)	0 (0.0)	0 (0.0)	0 (0.0)
monobactam	1 (5.0)	0 (0.0)	0 (0.0)
cephalosporin (first generation)	0 (0.0)	0 (0.0)	2 (8.0)
miscellaneous^b^	0 (0.0)	1 (2.6)	1 (4.0)
glycopeptide	7 (35.0)	15 (38.5)	5 (20.0)
oxazolidinone	0 (0.0)	0 (0.0)	1 (4.0)
other^c^	0 (0.0)	0 (0.0)	0 (0.0)

a Antimicrobials used as monotherapy or in combination with other agents.

b Fosfomycin, metronidazole, nitrofurantoin, trimethoprim/sulfamethoxazole.

c Azithromycin, clindamycin, daptomycin, doxycycline, fluconazole, micafungin, tigecycline.

d Sepsis, fever (neutropenic, post-operative, unknown origin), osteomyelitis, prosthetic joint infection, leucocytosis.

**Figure 1.**
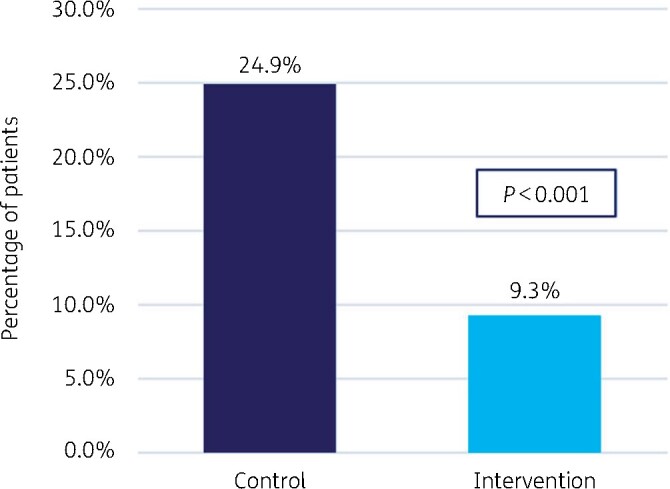
Overall rate of 30 day readmission.

Our analysis showed a significant reduction in overall readmission rates for patients whose antibiotic therapies underwent ASP interventions as compared with the historical control cohort: 29 versus 135 patients (9% versus 24.9%; *P *<* *0.001) (Figure 1).

**Figure 2.**
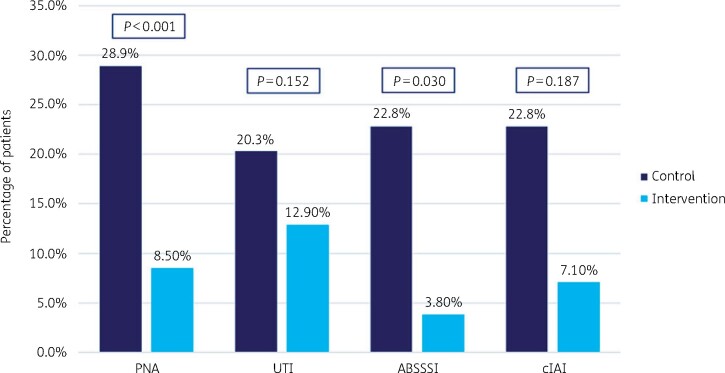
Rate of 30 day readmission by infection type.

The readmission rates for patients diagnosed with PNA and ABSSSI were significantly lower in the ASP-intervention group as compared with the historical control group: 11 versus 77 patients (8.5% versus 28.9%; *P *<* *0.001) and 1 versus 19 (3.8% versus 22.8%; *P *=* *0.03), respectively. We identified a difference in the readmission rates for UTI and cIAI between the ASP-intervention cohort and the historical control group as well. However, the decrease did not reach statistical significance: 13 versus 27 patients (13% versus 20.3%; *P *=* *0.125) and 1 versus 14 (7.1% versus 22.8%; *P *=* *0.187), respectively (Figure 2).

Of note, the 30 day readmission rate was significantly lower for patients diagnosed with CDI in the ASP-intervention group as compared with the historical control group, 1 versus 13 (0.3% versus 2.4%; *P *=* *0.025).

ASP interventions did not lead to higher in-hospital mortalities as compared with the control cohort. To the contrary, our analysis suggested that the mortality for the intervention group decreased significantly to 16 versus 52 patients (5.4% versus 9.6%; *P *=* *0.033).

We did not demonstrate a difference in the LOS between the ASP-intervention and historical control groups, 7.26 days versus 7.5 days (*P *=* *0.21).

Our ASP programme resulted in a dramatic reduction in antibiotic expenditure. Over a 6 month period, between January and June 2017, the expenditure per adjusted patient days (APD) decreased to $4.37 as compared with $23.33 for the historical control group. This translated into a total cost for antibiotics during the control period of $379 643 as compared with $67 721 during the ASP-intervention period. The discrepancy in cost suggests the uninhibited administration of antibiotics in the historical control group as compared with the antibiotic usage after ASP intervention (Table 1).

## Discussion

Studying the association between ASP efforts and patient outcomes is of great importance as it increases our understanding of how ASPs contribute to the patient’s overall quality of care. We show that a stringent ASP can be safely implemented in an elderly hospitalized patient population without discernible adverse outcomes. Specifically, rigorous ASP interventions in patients older than 65 years correlated with an overall reduction in 30 day readmissions in patients for all studied infection types. This is remarkable as several past studies did not find an association between readmissions and ASPs.^11^^,^^12^^,^^16–18^ Moreover, Ritchie *et al*.^13^ suggested that antibiotic treatment recommendations may actually have contributed to an increase in readmissions while it decreased the LOS in patients with cellulitis. We cannot readily explain this difference as the correlations between different outcome measures are complex and vary amongst hospitals and patient populations.^19^ However, Chopra *et al.*^20^ showed a correlation between decreasing CDIs and decreasing readmission rates, which may have contributed to the decrease in readmissions in our study. Moreover, our ASP efforts did not affect the LOS of our intervention group. This could have furthermore decreased readmission rates, as we know that a decrease in LOS may adversely affect readmissions.^12^^,^^13^

Interestingly, we observed a statistically significant decrease in in patient mortality in patients whose antibiotic treatments were monitored by the ASP, underscoring the safety of the program.^21–23^ This is an important finding as narrowing and discontinuation of antibiotic therapies can be challenging because of a fear for the ‘wellbeing of patients’, particularly in a vulnerable, elderly population. Ritchie *et al*.,^13^ similarly, demonstrated a decrease in mortality in their patient population that underwent antibiotic treatment recommendations. Additional studies underscore the safety of ASPs by suggesting no negative effects on mortality.^12^^,^^24–26^

Finally, we demonstrate a significant decrease in antibiotic expenditure per APD. Substantial cost savings have been demonstrated by other investigators and underscore the cost-saving opportunities that ASPs offer.^11^^,^^13^^,^^19^ Indeed, our savings formed the basis to employ our Clinical Pharmacist permanently, full-time. In the current healthcare environment, convincing healthcare administrators to invest in additional full-time equivalents requires solid evidence of their contribution to safety and quality, but also demonstration of financial viability.

We have identified limitations to our study as the levofloxacin usage differed significantly between the historical control group and the intervention group. FDA warnings against the use of fluoroquinolone antibiotics likely contributed to this difference.^27^ We also noticed an overall greater administration of broad-spectrum antibiotics in the historical control group. The greater number of cIAI in our historical control group could have contributed to this difference. Most importantly, we launched our ASP in the fourth quarter of 2015. Therefore, antibiotic treatment interventions were conducted during the time period between the historical control group and the ASP intervention group. This undoubtedly contributed to the greater usage of narrow-spectrum antibiotics and decreased utilization of fluoroquinolones in the ASP intervention group. Although this may have accentuated our results it should not have diminished the effect of the ASP on the studied quality metrics. Further limitations of cohort studies include an inherent selective bias: more seriously ill patients receive broader antibiotic therapies as compared with less ill individuals. We did not apply a severity score to the intervention and control group confounding the possibility of a bias.

Despite the apparent limiting factors of this study, or results suggest that rigorous ASPs can be safely and successfully implemented in elderly, hospitalized patients.

Future research needs to be directed towards ensuring standardization and unequivocal reproducibility of ASP interventions to optimize patient outcomes and allow comparisons between different healthcare settings.

## Funding

This study was carried out as part of our routine work.

## Transparency declarations

None to declare.

### Author contributions

R.A.T., J.M. and S.K. collected the data for this study. R.A.T., J.M., S.K. and D.L. summarized the data. R.A.T. wrote the first draft of this manuscript. All authors, including R.A.T., J.M., S.K., D.L. and J.P. contributed to the interpretation of the findings, critically reviewed subsequent revisions and approved the final version.
